# 

**Published:** 1853-07

**Authors:** 


*N. Y. Med. Times.*—Our thanks are due to the editor of the New York Med. Times for a copy of the proceedings of the Am. Med. Association, issued promptly after the adjournment of the meeting. Our notice of the proceedings was prepared before this copy was received, but we are not the less obliged, on that account, for the courteous attention.

---

☞ Our readers will notice that the contents of this No. consist entirely of original matter. Several articles selected for the eclectic department are deferred.

An article by Prof. C. B. Coventry, on the late meeting of the Am. Med Association, will appear in our next issue.

The report on Dysentery, commenced in this No., will be continued.

